# Child maltreatment and pediatric pain: A survey of healthcare professionals’ pain knowledge and pain management techniques

**DOI:** 10.1177/13674935231167965

**Published:** 2023-04-05

**Authors:** Sarah Campbell, Matthew Baker, Kelly McWilliams, Shanna Williams

**Affiliations:** 1 Department of Educational and Counselling Psychology, 5620 McGill University, Montreal, QC, Canada; 2 Graduate Center and John Jay College of Criminal Justice, 2009 City University of New York, New York, NY, USA

**Keywords:** pain, pediatrics, child abuse, evidence-based practice, interview

## Abstract

Children who have been maltreated are at an increased risk of having their pain under-recognized and undertreated by healthcare professionals, and thus, are more susceptible to adverse outcomes associated with undertreated pain. This study’s aims were to examine: (*1*) if healthcare professionals’ pediatric pain knowledge is associated with their pain assessment methods, (*2*) if maltreatment-specific pain knowledge is associated with consideration of child maltreatment when deciding on a pain management strategy, and (*3*) if pediatric pain knowledge would relate to maltreatment-specific pain knowledge. A sample (*N* = 108) of healthcare professionals responded to a survey designed to examine their current knowledge and utilization of pediatric pain assessment and management with emphasis on the effects of child maltreatment. Findings revealed healthcare professionals’ knowledge of pediatric pain is independent of their pain assessment and management practices. However, general pain knowledge was associated with maltreatment-specific pain knowledge and generally, healthcare professionals were knowledgeable of child maltreatment’s impact on pediatric pain. Participants who considered a history of maltreatment were also more likely to employ sensitive questioning strategies when asking children about their pain.

## Introduction

It has been estimated that upwards of 32% of adults have experienced some form of abuse as children (Afifi et al., 2014). Importantly, children who have experienced maltreatment are more susceptible to numerous adverse consequences including various psychiatric disorders (Teicher and Samson, 2013), depression (Humphreys et al., 2020), substance use disorders (Afifi et al., 2012), and poor emotion regulation (Gruhn and Compas, 2020). Additionally, children who have experienced maltreatment are more likely to experience pain later in life (Failo et al., 2019; Jones et al., 2009; Sachs-Ericsson et al., 2007; Tsur et al., 2020) and the number of transgressions a child experiences has a cumulative effect on adult chronic pain (Brown et al., 2018).

Pain is also central to a child’s experience of abuse and is frequently mentioned during children’s legal testimonies (Tsur et al., 2020). Some research has even traced early life stressors’ effects, including trauma, on nociceptive circuits, which increase one’s vulnerability to chronic pain (Burke et al., 2017). As such, pain is a core feature of maltreatment with residual effects, contributing to a child’s suffering and pathological development and thus, must be accurately assessed and managed.

To date, little research has examined links between child maltreatment and pediatric pain in healthcare contexts. As a result, the current study aims to address this research gap by examining medical professionals’ knowledge of child maltreatment and pain, with a specific focus on their knowledge of pain assessment in maltreated pediatric populations.

### Pain in pediatric populations

Pain in general pediatric populations is prevalent in healthcare systems (Taylor et al., 2008). Approximately one-third of children in Canadian hospitals experience moderate to severe levels of pain (Stevens et al., 2012) and over two-thirds of children undergo a painful procedure every 24 h (Stevens et al., 2011). Despite high prevalence rates of pain, studies have found that pediatric pain is inadequately assessed and mismanaged in children’s hospitals (Birnie et al., 2014). Best-practices incorporate multiple sources of information, such as behavioral cues, in conjunction with validated self-report measures to gauge a child’s pain level (Von Baeyer, 2006). However, approximately 60% of pediatric pain assessments in Canadian hospitals are preformed using a single, non-validated measure, such as unspecified behavioral scales or checking a box to indicate that pain is present or absent (Stevens et al., 2012) raising concerns about how well a child’s experience is captured.

Insufficient pain assessment is concerning because mismanaged pediatric pain is associated with numerous adverse effects in addition to patient distress (Oddson et al., 2006; Palermo, 2000; Roth-Isigkeit, 2005). Broadly, pediatric pain has been associated with lower quality of life (Oddson et al., 2006) and negatively impacts children and their family’s daily functioning (Palermo, 2000). Thus, it is critical for healthcare professionals to use evidence-based pain assessment strategies in order to accurately recognize and estimate pain in pediatric populations and prescribe appropriate management strategies.

### Pain assessment

Healthcare professionals’ assessments are influenced by a multitude of top-down and bottom-up processes (Craig et al., 2010; Goubert et al., 2005). Top-down influences include observers’ beliefs, experiences, knowledge, and pain education, while bottom-up influences consist of external factors such as patients’ pain expression and contextual cues (Goubert et al., 2005). This raises concern for children who have been maltreated as both top-down and bottom-up influences appear to be compromised. Top-down influences have an important role as research has suggested that a lack of knowledge contributes to improper pain assessment and management (Hroch et al., 2019). For example, interventions emphasizing *education, reminders,* and *feedback* on proper pain assessment have been found to improve assessment (Zhu et al., 2012). However, these programs vary in quality and focus on improving pain assessment without addressing those who have experienced maltreatment (Gagnon et al., 2016). Thus, without research investigating healthcare professionals’ knowledge of maltreatment and pain, there is a risk that top-down influences of pain assessment for maltreated populations may be compromised.

Bottom-up factors also influence observers’ pain assessment ratings. For example, Craig et al. (2010) found that patients’ pain expression significantly impacts observers’ pain ratings and automatic empathetic reactions. Concerningly, children in general populations have been found to suppress their pain more often than they exaggerate it (Larochette et al., 2006) and children with a history of maltreatment might be at particular risk as they have been found to suppress their emotional expression in a variety of settings, such as in forensic settings (Karni-Visel et al., 2019) and medical settings (Drouineau et al., 2017). A corollary of such emotional suppression may be underestimated pain, as demonstrated by Drouineau et al. (2017) who found that healthcare professionals underestimated maltreated children’s pain.

There is a dearth of research examining healthcare professionals’ knowledge of maltreatment’s impact on pain expression and assessment, and whether healthcare professionals tailor their pain assessment and management techniques for maltreated children. Employing efficacious pain assessment and management techniques is fundamental for sufficient care, especially for maltreated populations. The current study investigated healthcare professionals' knowledge of pediatric pain and child maltreatment, as well as their current pain assessment and management techniques.

### Aims

This study had three aims. First, we aimed to examine healthcare professionals’ frequency of using validated pain assessment measures and factors associated with their use. Second, we aimed to examine whether healthcare professionals consider a history of maltreatment when assessing pain and deciding on a pain management strategy. Lastly, we aimed to examine a relationship between healthcare professionals’ knowledge of pain, child maltreatment, and knowledge of associations between pain and child maltreatment.

## Method

### Procedure

Participants were recruited from multiple sites across Canada and the United States to complete a survey through relevant professional and hospital association emails and newsletters, Prolific, and snowball sampling from April 2021 to February 2022. Participants received a $15 Amazon gift card as compensation for study participation. In order to participate, respondents needed to be actively working healthcare professionals and be above the age of 18. This study was approved by the McGill University research ethics board.

### Measures

A survey was developed to examine healthcare professionals’ methods of pain assessment and management, as well as knowledge of pediatric pain and maltreatment. The first section assessed participants’ methods of pain assessment and consisted of self-report rating questions and application-based questions. Questions asked healthcare professionals to report on factors they consider and which methods (e.g., assessment tools, types of questions, etc.) they use when assessing children’s pain. For example, participants’ frequency of using supportive, non-suggestive statements was measured on a 3-point Likert scale, as *Always* (1), *Sometimes* (2), and *Never* (3). Participants were also asked *yes* or *no* questions about whether they consider a history of maltreatment when assessing children’s pain and selecting a pain management strategy. Questions evaluating which validated pain assessment measures healthcare professionals most frequently used were based on (Stinson et al., 2006) review of common single-item pain intensity measures and (Stevens et al., 2012) investigation of pain assessment for hospitalized children in Canada. These measures included the Pieces of Hurt Tool (Hester, 1979), faces scales (e.g., Wong-Baker FACES Pain Scale [Garra et al., 2010]; Faces Pain Scale-Revised [Hicks et al., 2001]); Visual Analogue Scales (Langley and Sheppeard, 1985), and the Numerical Rating Scale (Von Baeyer et al., 2009). The full survey is available in the Supplemental Materials.

The questionnaire’s second part examined healthcare professionals’ knowledge of pediatric pain and was based on the *Knowledge and Attitudes Survey* (Ferrell and McCaffery, 2014) and *Pediatric Nurses' Knowledge and Attitudes Survey Regarding Pain* (Manworren, 2001); both previously validated questionnaires used to measure healthcare professions’ knowledge and attitudes towards pediatric pain. Relevant items from the original questionnaires were kept and new questions about child maltreatment were added to obtain a measure of participants’ knowledge about child maltreatment.

### Statistical analysis

Descriptive statistics were used to report healthcare professionals’ frequency of using pediatric pain assessment measures. A linear regression was used to examine the first hypothesis. That is, based on prior research (Pouralizadeh et al., 2021; Zhu et al., 2012), we hypothesized that healthcare professionals with higher knowledge levels of pediatric pain would use evidence-based measures more frequently than healthcare professionals with lower levels of pediatric pain knowledge. *Total Knowledge Accuracy* scores were calculated as a continuous variable based on the percentage of correctly answered questions out of the 28 questions from the knowledge section of the questionnaire. *Use of Measures* scores were calculated as a continuous variable by averaging healthcare professionals’ reported frequency of using the four validated measures, which they rated as *Never* (1), *Sometimes* (2), and *Often* (3).

A bivariate logistic regression was used to test the second hypothesis that healthcare professionals with greater maltreatment-specific pain knowledge would be more likely to consider a history of maltreatment when deciding on a pain management strategy. The predictor variable, *Maltreatment Knowledge Accuracy* scores, were calculated using the percentage of correct responses from the 10 knowledge questions about maltreated populations. The outcome variable was measured from binary responses (i.e., *yes* or *no*) to the question “Do you consider a history of maltreatment when deciding on a pain management strategy for a child?”

Lastly, a linear regression was used to test the final hypothesis, in which we postulated that healthcare professionals who exhibited greater knowledge of pediatric pain would also be more likely to exhibit greater knowledge of child maltreatment and its’ effects on children’s pain. Exploratory statistics were conducted to further test for a relationship between healthcare professionals' knowledge and consideration of maltreatment, and their pain assessment and management practices. All statistical analyses were conducted using IBM SPSS version 28 (IBM Corporation, 2021). Anonymized data are available upon request from the corresponding author.

## Results

### Participants

One hundred and eight healthcare professionals from across Canada and the United States completed the survey. Complete demographic information can be found in Table 1. Saliently, 15 (13.9%) healthcare professionals reported receiving continuing education on pain and 16 (14.8%) reported receiving continuing education on maltreatment or trauma’s effects on pain.

**Table 1.** Demographic characteristics of participants.

Variable	*n (%)*
Age (range)	
18–24	13 (12.0)
25–34	40 (37.0)
35–44	28 (25.9)
45–54	16 (14.8)
55–64	7 (6.5)
65–74	3 (2.8)
75–84	1 (0.9)
Gender	
Male	21 (19.4)
Female	87 (80.6)
Profession^a^	
Medical doctor	31 (28.7)
Registered nurse	54 (50.0)
Other	21 (19.4)

*Note:* Demographic information is presented for the overall sample about participants’ age, gender, and profession.

^a^Two participants chose not to respond.

### Missing data

Missing data analysis revealed that 8.85% of responses were missing at the item level. Given that less than 10% of the data are missing, it was omitted on case-wise basis. The sample size included in each analysis is reported when the analysis is presented.

### Knowledge and practice

Health care professionals’ mean total knowledge accuracy was 74.19% (*Standard Deviation* = 15.40) and mean maltreatment knowledge accuracy was 74.11% (*Standard Deviation* = 17.37). Numerical Rating Scales (Von Baeyer et al., 2009) were most frequently used among healthcare professionals (95.3%, *n* = 102), followed by faces scales (87.9%, *n* = 94; e.g., Wong-Baker FACES Pain Scale [Garra et al., 2010]; Faces Pain Scale [Hicks et al., 2001]), Visual Analogue Scales (72.9%, *n* = 78; Langley and Sheppeard, 1985), and Pieces of Hurt Tool (26.4%, *n* = 28; Hester, 1979). All but one participant (99.1%; *n* = 105 of 106 participants who responded to the question) reported asking children directly about their pain and 103 (96.3% of 107 participants who responded to the question) reported asking children’s caregivers about their child’s pain.

Participants were asked if they assess chronic pain (i.e., pain in a non-urgent context), and of those who indicated that they do (75.9%; *n* = 82), 95.1% (*n* = 78) reported that they do consider if a child has a history of maltreatment while assessing their pain. Seventy-seven (93.9% out the 82 respondents) of these healthcare professionals who consider a history of maltreatment while assessing pain also considered that maltreated children may communicate their pain differently than non-maltreated children; however, three (3.7% out of the 82 respondents) out of the four participants who do not consider a history of maltreatment while assessing pain considered that maltreated children may communicate their pain differently than non-maltreated children.

Ninety-two (89.3% of 103 participants who responded to the question) participants reported considering a history of maltreatment when deciding on a pain management strategy for a pediatric patient. One hundred two (94.4%) healthcare professionals reported on whether they consider a history of maltreatment when deciding on a pain management strategy and if maltreated children may communicate their pain differently than non-maltreated children. Eighty-nine (87.3% out of 102 respondents) healthcare professionals who consider a history of maltreatment while deciding on a pain management strategy also considered that maltreated children may communicate their pain differently than non-maltreated children; however, nine (8.8% out of 102 respondents) out of eleven participants who do not consider a history of maltreatment when deciding on a pain management strategy did report considering that maltreated children may communicate their pain differently than non-maltreated children.

### Hypothesis testing

The model examining healthcare professionals’ frequency of using validated pain assessment measures as a function of their knowledge of pediatric pain was not significant (*B* = −0.09, *p* = .352). Coefficients from the analysis are presented in Table 2.

**Table 2.** Results of linear regression analyses TKA scores predicting use of measures scores and MKA scores.

Variable	Use of measures scores				MKA score			
	Unstandardiz-ed *B*	Standardized beta ( β )	Standard error	*p*-value	Unstandardiz-ed *B*	Standardized beta ( β )	Standard error	*p*-value
TKA score	−.09	−.09	.10	.352	.84	.72	.08	<.001*
*R* ^*2*^	.01				.52			

*Note:* Table outlines results of the linear regression analyses that were used to (*1*) model the relationship between Total Knowledge Accuracy scores and Use of Measures scores, and (*2*) model the relationship between Total Knowledge Accuracy scores and Maltreatment Knowledge Accuracy scores. TKA - Total Knowledge Accuracy; MKA - Maltreatment Knowledge Accuracy. *Significant result.

The model examining healthcare professionals’ knowledge of child maltreatment as a function of their consideration of child maltreatment when developing a pain management strategy was not significant, χ2 (1, *N* = 108) = 0.69, *p* = .407.

Regression analysis investigating hypothesis three, the association between healthcare professionals’ total knowledge scores and their maltreatment knowledge scores, was found to be significant (*B* = 0.77, *p* < .001; see Table 2).

Exploratory analysis revealed a significant difference between healthcare professionals who consider a history of maltreatment and those who do not (when deciding on a pain management strategy) in their frequency of using supportive, non-suggestive statements when assessing children’s pain, *t* (11.30) = −4.20, *p* = .001, Cohen’s *d* = 1.34, 95% CI [0.66,2.01]. Healthcare professionals who reported considering a history of maltreatment also reported using supportive, non-suggestive statements more frequently (*Mean*_*supportive, non-suggestive statements*_ = 1.94) than healthcare professionals who reported they did not consider a history of maltreatment (*Mean*_*supportive, non-suggestive statements*_ = 2.66). Upon examination of the proportion of healthcare professionals (out of 102 who responded to both questions) who consider a history of maltreatment when deciding on a pain management strategy and whether they use supportive, non-suggestive statements, it was found that 1.0% of participants (*n* = 1) *never* use supportive, non-suggestive statements, 28.4% (*n* = 29) *sometimes* use supportive, non-suggestive statements, and 60.8% (*n* = 62) *often* use supportive, non-suggestive statements when assessing a child in pain. Ten participants (out of 102 responding to both questions) reported that they do not consider if a child has a history of maltreatment when deciding on a pain management strategy, and one healthcare professional (1.0% of the overall sample) reported *never* using supportive, non-suggestive statements, 7.8% (of the overall sample; *n* = 8) reported *sometimes* using supportive, non-suggestive statements, and one (1.0% of the overall sample) reported *often* using supportive, non-suggestive statements when assessing a child in pain.

## Discussion

This study’s purpose was to investigate healthcare professionals’ knowledge of pain in maltreated and non-maltreated pediatric populations, as well as healthcare professionals’ pain assessment and management methods. We achieved our three aims, the first of which was to examine healthcare professionals’ frequency of using validated pain assessment measures and factors associated with their use. The second examined whether healthcare professionals consider a history of maltreatment when assessing pain and deciding on a pain management strategy, and the third examined healthcare professionals’ knowledge of pain and child maltreatment as well as the associations between pain and child maltreatment. It was expected that (*1*) knowledge of pain would be positively correlated to more frequent use of validated assessment measures, (*2*) that healthcare professionals with higher levels of knowledge regarding maltreatment’s impact on pain will be more likely to consider a history of maltreatment when deciding on a pain management strategy, and (*3*) that healthcare professionals total knowledge accuracy and maltreatment knowledge accuracy scores would be positively correlated. Results for the first two hypotheses were found to be nonsignificant, suggesting that healthcare professionals’ knowledge of pain is independent of their pain assessment (i.e., frequency of using validated pain assessment measures) and management methods (i.e., considering a history of child maltreatment when developing pain management strategies). However, results for the third hypothesis were supported, suggesting that healthcare professionals with higher levels of general pain knowledge, also have higher levels of maltreatment-specific knowledge.

Our study’s results suggest that almost all healthcare professionals reported considering maltreatment history in some way when assessing and managing pediatric pain. Surprisingly, however, very few healthcare professionals received any continuing education on pain (*n* = 15) or on maltreatment’s impact on pain (*n* = 16). Additionally, the mean total knowledge accuracy score in this study was 74.19%, indicating slightly greater knowledge scores than Hroch et al.’s prior findings (*Mean* = 67%, *Standard Deviation* = 9.1) which suggested that knowledge of pain is low among healthcare professionals (Hroch et al., 2019). This difference could be explained by adaptations made to the original Knowledge and Attitudes Survey for our study’s purposes. Hroch et al. (2019) reported that questions regarding medication administration were an area of low knowledge; however, because this was not an area of interest for our study, several of these questions were removed or replaced with maltreatment specific questions.

The nonsignificant results of our study may also be explained by knowledge translation research, which suggests that there is a disconnect between one’s level of knowledge and their implementation of this knowledge in practice (Zhu et al., 2012). Thus, increased knowledge of pain does not necessarily equate to proper pain assessment and management and vice-versa. In our study’s case, it appears that despite very few participants having received continuing education on maltreatments’ impact on pain, almost all healthcare professionals reported considering maltreatment in their practice.

A salient finding was that healthcare professionals who reported using supportive, non-suggestive statements more frequently during their assessment of pediatric pain were significantly more likely to consider a history of maltreatment when deciding on a pain management strategy. Supportive, non-suggestive statements validate a child’s experience without suggesting a specific or preferred response (e.g., thanking a child for helping a clinician understand how they are feeling) and have been found to be beneficial when working with maltreated children in other settings (Hershkowitz and Lamb, 2020). This suggests that aspects of healthcare professionals’ practice may be associated with one another as some healthcare professionals might be more likely to use specific methods or strategies.

Our study’s results about healthcare professionals’ most frequently used pain assessment measures is consistent with Stevens et al. (2012)’s findings, where the Numerical Rating Scale (Von Baeyer et al., 2009) is most commonly used, and the Pieces of Hurt Tool (Hester, 1979) one of the least frequently used.

### Limitations

The study’s sample size was relatively small and as a result may not have been representative of the overall healthcare professional population. Additionally, participant recruitment relied on self-selection, whereby participants were individuals who were willing to complete a voluntary questionnaire.

Healthcare professionals’ assessment methods were determined through self-report. Participants’ responses may have been influenced by various forms of bias including social desirability bias or recall bias and may have not reflected their day-to-day practice. Although our study indicates that many healthcare professionals report considering maltreatment history in their practice, our study did not collect data on *how* they consider it (e.g., how they adapt their assessment methods or management techniques). Similarly, individuals may differ in what they consider to be using a measure as *often*, meaning that the frequency of using validated measures is not standardized between participants. There also appeared to have been a ceiling effect for the questionnaire’s knowledge portion, thereby reducing the variability in healthcare professionals’ pain knowledge scores.

Some research has found that validated measures are misused, such as measures being used with age-inappropriate groups (Stevens et al., 2012). For example, younger children have limited language and cognitive abilities and thus, pain measures developed for older children may be misunderstood by younger populations (Merkel and Malviya, 2000). Although our study examined how frequently healthcare professionals use validated pain assessment measures, it did not assess if these measures were used appropriately, which is a further limitation of the survey’s self-report methodology. Stinson et al. (2006) found that no single validated measure is optimal for all patients and situations, therefore, healthcare professionals must understand how to adapt their assessment methods.

### Implications for practice

Our study indicates that the majority of healthcare professionals report considering maltreatment history when dealing with pediatric patients’ pain. Furthermore, healthcare professionals appear to have some knowledge of child maltreatment’s impact on pediatric pain; however, pain in Canadian children’s hospitals continues to be undermanaged (Birnie et al., 2014; Stevens et al., 2012). As such, it is important to investigate *how* healthcare professionals are considering child maltreatment when they assess pain and when they adapt their pain management methods. It is promising that healthcare professionals are aware of maltreatment’s impact on pain during their practice; however, they should continue to be supported in implementing specific strategies to target children who have been maltreated. Similarly, research should work to develop accurate assessment strategies for children with either a known or suspected history of maltreatment. Clinicians should continue to be taught valuable skills, including techniques aimed at eliciting more detailed pain narratives through rapport building and compassionate questioning. Lastly, implementing trauma-informed care is critical as a child’s developmental history, including a history of maltreatment and other trauma, may not be known to an attending healthcare professional.

## Conclusion

Results from this study provide insight to healthcare professionals’ knowledge and consideration of child maltreatment when treating pediatric pain. Our results suggest that healthcare professionals’ knowledge of pediatric pain is independent of their pain assessment and management methods. Therefore, future strategies aimed at improving healthcare professionals’ pain assessment methods should move beyond improving knowledge and work to develop specific strategies aimed at supporting healthcare professionals’ adapt their pain evaluation methods. Findings also revealed that healthcare professionals’ consideration of child maltreatment history was associated with compassionate questioning strategies. This raises the question, “what modulates this difference in healthcare professionals’ practice, if not knowledge?” Future research should continue to investigate how maltreatment is considered in medial settings as well as associations between maltreatment and pediatric pain communication.

## Supplemental Material

Supplemental Material - Child maltreatment and pediatric pain: A survey of healthcare professionals’ pain knowledge and pain management techniques Supplemental Material for Child maltreatment and pediatric pain: A survey of healthcare professionals’ pain knowledge and pain management techniques by Sarah Campbell, Matthew Baker, Kelly McWilliams and Shanna Williams in Journal of Child Health Care
