# Supplementary material for: Child maltreatment and pediatric pain: A survey of healthcare professionals’ pain knowledge and pain management techniques
Source: J Child Health Care. 2023 Apr 5;28(4):774–85. doi: 10.1177/13674935231167965 (PMC11607853; doi:10.1177/13674935231167965)
Supplement: Supplemental Material - Child maltreatment and pediatric pain: A survey of healthcare professionals’ pain knowledge and pain management techniques [file sj-pdf-1-chc-10.1177_13674935231167965.pdf]

Supplemental Materials Titles and Descriptions

Title

Survey - Supplemental material for: Child maltreatment and pediatric pain: A survey of healthcare professionals' pain knowledge and pain management techniques

Description

Supplemental material, Survey, for: Child maltreatment and pediatric pain: A survey of healthcare professionals' pain knowledge and pain management techniques by Campbell, Baker, McWilliams, & Williams in Journal of Child Health Care

Supplemental Material: Survey

Pain Questionnaire

Start of Block: Demographics

Q2 What is your age?

▼ 18 - 24 (1) ... I do not want to respond (9)

Q3 What gender do you identify as?
o Male (1)
o Female (2)
o Non-binary (3)
o Prefer not to say (4)
o Other (5) ____
o I do not want to respond (6)

Q4 Which province/territory or state do you work in?

▼ Alberta (Canada) (1) ... I do not want to respond (111)

Q5 Do you practice your profession in an urban or rural setting?
o Urban (1)
o Rural (2)
o I do not want to respond (3)

Q6 What is your profession? Please specify (e.g., paediatrician)
o Medical doctor (1) ____
o Registered Nurse (2) ____
o Other (3) ____
o I do not want to respond (4)

Q7 Number of years working in the field

▼ Under 1 year (1) ... I do not want to respond (7)

Q8 Do you work with children (or adolescents under the age of 18)?
o Yes (1)
o Sometimes (2)

o No (3)
o I do not want to respond (8)

Display This Question:

If Do you work with children (or adolescents under the age of 18)? != No

Q9 What age range of children do you most frequently work with?

____

Display This Question:

If Do you work with children (or adolescents under the age of 18)? != No

Q10 Do you work with children (or adolescents under the age of 18) who are in pain?
o Yes (1)
o Sometimes (2)
o No (3)
o I do not want to respond (4)

Display This Question:

If Do you work with children (or adolescents under the age of 18) who are in pain? != No

Q11 What type of pain are the children that you work with typically experiencing? (select all that apply)
▢ Physical pain (1)
▢ Emotional/psychological pain (2)
▢ Acute pain (3)
▢ Chronic pain (4)
▢ I do not want to respond (5)

Q12 Do you have children of your own?
o Yes (1)
o No (2)
o I do not want to respond (3)

Q13 Have you taken any continuing education on pediatric pain? If yes, please describe.
o Yes (1) ____
o No (2)
o I do not want to respond (3)

Q14 Do you have experience working with Youth Protection or Child Protective Services?

o Yes (1)
o No (2)
o I do not want to respond (3)

Q15 Have you taken any continuing education on how developmental trauma and/or having a history of childhood maltreatment might influence pediatric pain assessment (i.e., physical abuse, sexual abuse, emotional abuse, or neglect)? If yes, please describe.
o Yes (1) ____
o No (2)
o I do not want to respond (3)

End of Block: Demographics

Start of Block: Pain Assessment

Q16 In this portion of the survey we will be asking you to reflect on your experiences working with children in medical settings who are experiencing pain. We would like to know about your experience assessing pain in pediatric populations.

Q17 Please rate how often you use each method when assessing pain in children.

	Frequency			
	Never (1)	Sometimes (2)	Often (3)	I do not want to respond (4)
Pieces of Hurt Tool (e.g., poker chip) (1)	o	o	o	o
Faces scales (e.g., Baker-Wong FACES) (2)	o	o	o	o
Visual Analogue Scales (i.e., indicating pain intensity on a visual line) (3)	o	o	o	o
Numerical rating scales (e.g., scale of 1 - 10) (4)	o	o	o	o
Asking the child directly (5)	o	o	o	o

Asking the child's caregiver (6)	o	o	o	o
Other: (7)	o	o	o	o

Q18 In medical settings, what is the best way to ask children about **whether they are experiencing any pain**? (select all that apply)

▢ Open-ended questions (i.e., tell me how you are feeling) (1)
▢ Option-posing questions (i.e., yes/no questions; does it hurt?) (2)
▢ Standardized measures (e.g., number scale, faces scale) (3)
▢ All of the above (4)
▢ None of the above (5)
▢ I do not want to respond (6)

Q19 In medical settings, what is the best way to ask children **about their pain level** (severity of the pain)? (select all that apply)

▢ Open-ended questions (i.e., tell me how you are feeling) (1)
▢ Option-posing questions (i.e., yes/no questions; does it hurt?) (2)
▢ Standardized measures (e.g., number scale, faces scale) (3)
▢ All of the above (4)
▢ None of the above (5)
▢ I do not want to respond (6)

Q20 When asking children about their pain, do you concentrate on the manner in which you ask them questions about their pain? That is, do you explicitly consider the type of questions you are asking (e.g., open-ended vs. closed-ended)?

o No (1)
o Sometimes (2)
o Often (3)
o Not sure (4)
o I do not want to respond (5)

Q21 When judging **if a child is in pain**, please rate the factors you take into account.

	Frequency			
	Never (1)	Sometimes (2)	Often (3)	I do not want to respond (4)
Self-report (1)	o	o	o	o

Parent/guardian report (2)	o	o	o	o
Physiological measures (i.e., vital signs) (3)	o	o	o	o
Paralinguistic signs (i.e. groaning, moaning) (4)	o	o	o	o
Other behavioural cues (i.e., guarding) (5)	o	o	o	o
Other (6)	o	o	o	o

Q22 When judging **the severity** of a child's pain, please rate the factors you take into account.

	Frequency			
	Never (1)	Sometimes (2)	Often (3)	I do not want to respond (4)
Self-report (1)	o	o	o	o
Parent/guardian report (2)	o	o	o	o
Physiological measures (i.e., vital signs) (3)	o	o	o	o
Paralinguistic signs (i.e. groaning, moaning) (4)	o	o	o	o
Other behavioural cues (i.e., guarding) (5)	o	o	o	o
Other (6)	o	o	o	o

Q23 Do you use supportive non-suggestive statements when assessing a child's pain? Supportive non-suggestive statements are statements that validate a child’s experience without suggesting a specific or preferred response (e.g., “thank you for helping me understand how you are feeling”).
o Always (1)
o Sometimes (2)
o Never (3)
o Not sure (4)
o I do not want to respond (5)

Q24 Generally, do you think children are:
o Honestly reporting their pain (1)
o Deceptively reporting their pain (2)
o Unsure (3)
o I do not want to respond (4)

Display This Question:

If Generally, do you think children are: = Deceptively reporting their pain

Q25 Do you think that children tend to exaggerate or conceal their pain?
o Exaggerate their pain (1)
o Conceal (or under report) their pain (2)
o I do not want to respond (3)

Q26 Do you assess children experiencing chronic pain (i.e., recurrent pain lasting three months or more) in your medical practice?
o Yes (1)
o No (2)
o I do not want to respond (3)

Display This Question:

If Do you assess children experiencing chronic pain (i.e., recurrent pain lasting three months or mo... = Yes

Q27 When assessing children’s chronic pain (i.e., recurrent pain lasting three months or more), do you consider if they have a history of maltreatment (i.e., abused)?
o Yes (1)
o No (2)
o I do not want to respond (3)

Display This Question:

If Do you assess children experiencing chronic pain (i.e., recurrent pain lasting three months or mo... = Yes

Q28 When assessing children’s chronic pain, do you consider if they have experienced any other developmental trauma (i.e., a parent with substance use difficulties, an incarcerated parent, a parent or sibling who has died etc.)?
o Yes (1)
o No (2)
o I do not want to respond (3)

Display This Question:

If When assessing children’s chronic pain (i.e., recurrent pain lasting three months or more), do yo... = Yes

Q29 Do you ask children who have a history of maltreatment (i.e., abused) about their pain differently than those who do not have a history of maltreatment? (i.e., through the use of empathic statements)
o Yes (1)
o No (2)
o I do not want to respond (3)

Display This Question:

If Do you ask children who have a history of maltreatment (i.e., abused) about their pain differentl... = Yes

Q30 How do you ask children who have a history of maltreatment about their pain differently than those who do not have a history of maltreatment? (Select all that apply)
▢ Through the use of empathic statements (1)
▢ Through prioritizing rapport building (2)
▢ Through providing more emotional support and reassurance (3)
▢ I do not want to respond (5)

Q31 When deciding on a management strategy for a child’s pain, do you consider if they have a history of maltreatment (i.e., abused)?
o Yes (1)
o No (2)
o I do not want to respond (3)

Q32 Do you consider that children who have a history of maltreatment may communicate their pain differently than children without a history of maltreatment?
o Yes (1)
o No (2)
o I do not want to respond (3)

Q33 In non-urgent settings, please rate how important you think it is to build rapport with a child prior to asking them about their pain.
o Extremely important (1)
o Very important (2)
o Moderately important (3)
o Slightly important (4)
o Not at all important (5)
o I do not want to respond (6)

Q34 How do you build rapport with a child? (select all that apply)
▢ Through small talk (e.g., asking their grade, favorite television show) (1)
▢ By asking them about a story (e.g., asking about their morning routine) (2)
▢ By playing a game with them (3)
▢ By telling them about yourself and your role in the medical setting (4)
▢ All of the above (5)
▢ Other (6) ____
▢ None of the above (7)
▢ I do not want to respond (8)

Q35 Are children more honest about their pain levels to medical professionals than other adults?
o Yes (1)
o No (2)
o Not sure (3)
o I do not want to respond (4)

End of Block: Pain Assessment

Start of Block: Scenario 1

Q36 **Please read the following scenario and answer the ensuing questions based on the information in the scenario.**

You are seeing Samantha, a 12 year old female, for a fractured clavicle. She is accompanied by her mother. Upon entering the room and greeting Samantha and her mother, you note that she appears to be guarding her clavicle and sitting very still. When you say “Hi” and introduce yourself she returns your greeting in a soft, friendly voice. She is relatively stoic, making few noises with a relatively normal facial expression, other than clenching her jaw when she moved once. Samantha’s mother seems concerned about her daughter’s level of pain and is asking for

something to be done about her pain. After greeting Samantha and her mother, you inquire about her pain.

Q37 Which of the following methods would you use to assess her pain? (select all that apply)
▢ Ask Samantha about her pain (i.e., in an interview style format) (1)
▢ Ask Samantha to rate her pain on a numeric scale (e.g., number rating scale) (2)
▢ Ask Samantha to rate her pain using a standardized rating tool (e.g., Faces Pain Scale-Revised) (3)
▢ Note Samantha's body language and facial expressions as cues to determine the severity of her pain (4)
▢ Note Samantha's autonomic measures (i.e., heart rate, blood pressure, respiratory rate) as a measure of the severity of her pain (5)
▢ Ask Samantha's mother what she thinks her pain level is (6)
▢ Other: (7) ____
▢ I do not want to respond (8)

Display This Question:

If Which of the following methods would you use to assess her pain? (select all that apply) = Ask Samantha about her pain (i.e., in an interview style format)

Q38 Considering the information provided in the scenario, which method below do you think would be the best way to ask if Samantha is in pain?
o You're pain, aren't you… how much does it hurt? (1)
o How much pain are you in? (2)
o Are you in pain Samantha? (3)
o Tell me how you are feeling Samantha? (4)
o Samantha, tell me how you are feeling in your body? (5)
o Other: (6) ____
o I do not want to respond (7)

Display This Question:

If Which of the following methods would you use to assess her pain? (select all that apply) = Ask Samantha to rate her pain using a standardized rating tool (e.g., Faces Pain Scale-Revised)

Q39 What standardized rating tool would be your first choice when assessing Samantha's pain?

____

Q40 Would you consider whether or not Samantha has a history of childhood maltreatment while **assessing** her pain?
o Yes (1)
o No (2)
o Depending on the context. (Please explain what context) (3)
____
o I do not want to respond (4)

Q41 Would you consider whether or not Samantha has a history of childhood maltreatment while **deciding on a pain management strategy** for her?
o Yes (1)
o No (2)
o Depending on the context. (Please explain what context) (3)
____
o I do not want to respond (4)

Q42 ***Scenario continued.***

Samantha's mother reveals that Samantha broke her collarbone during a fight at school with another girl who was making fun of her. Her mother goes on to share that Samantha has been having trouble at school recently as they only recently moved into an apartment. Prior to that, they lived in a women's shelter for approximately two months, as Samantha's mother found out that her ex-husband, Samantha's step-father, had been sexually abusing her.

Q43 Would you consider this information while assessing (and potentially when re-assessing) Samantha's pain?
o Yes (1)
o No (2)
o I do not want to respond (3)

Q44 Would you consider this information when deciding on a pain management strategy for Samantha?
o Yes (1)
o No (2)
o I do not want to respond (3)

Q45 Would you consider that Samantha's psychosocial history (e.g., family circumstances, life stressors, maltreatment history) may influence the way in which she communicates her pain?
o Yes (1)
o No (2)
o I do not want to respond (3)

End of Block: Scenario 1

Start of Block: Scenario 2

Q46 *Please read the following scenario and answer the ensuing questions based on the information in the scenario.*

Bruce, an 11 year old male, is seeking care for recurring headaches that vary in severity and frequent pain in his body, which varies in severity and location. Bruce is accompanied by his grandmother, his primary caregiver, as he was removed from his parents' care three years ago when it was found his parents had been neglecting him and physically abused him from the time he was 7 years old. His episodes of pain began approximately two years ago, although Bruce can't remember exactly when they started. They do, however, seem to be becoming more frequent and severe. Bruce's grandmother is concerned that his headaches and pain is affecting his sleep, and although Bruce has said he has trouble falling asleep on some nights, he is not sure if it is really affecting him. Bruce's grandmother discloses that sometimes he also says he is in significant pain but does not physically appear to be, which concerns her in that he may simply be looking for medication. However, other times he cries and screams because he is in so much pain, and it is during these episodes his grandmother reports he is inconsolable and nothing seems to make his pain better. Bruce's grandmother is worried about these episodes and unsure about how to help Bruce, but is also perplexed about the times that he does not show pain yet states that he is in pain.

Q47 Which of the following methods would you use to assess his pain? (select all that apply)

▢ Ask Bruce about his pain (i.e., in an interview style format) (1)

▢ Ask Bruce to rate his pain on a numeric scale (e.g., the number rating scale) (2)

▢ Ask Bruce to rate his pain using a standardized rating tool (e.g., the Faces Pain Scale) (3)

▢ Note Bruce's body language and facial expressions as cues to determine the severity of his pain (4)

▢ Ask Bruce's grandmother what she thinks his pain levels are (5)

▢ Other: (6) ____

▢ I do not want to respond (7)

Display This Question:

If Which of the following methods would you use to assess his pain? (select all that apply) = Ask Bruce about his pain (i.e., in an interview style format)

Q48 Considering the information provided in the scenario, which method below do you think would be the best way to ask Bruce about his pain currently?

o You’re in a lot of pain, aren’t you… how much does it hurt? (1)
o How much pain are you in? (2)
o Are you in pain Bruce? (3)
o Tell me how you are feeling Bruce? (4)
o Bruce, tell me how you are feeling in your body? (5)
o Other: (6) ____
o I do not want to respond (7)

Display This Question:

If Which of the following methods would you use to assess his pain? (select all that apply) = Ask Bruce about his pain (i.e., in an interview style format)

Q49 Considering the information provided in the scenario, which method below do you think would be the best way to ask Bruce about his pain from the previous week?
o You’ve been in a lot of pain, haven’t you… how many times in a week are you in pain? (1)
o Since the last time we saw each other, how have you felt? (2)
o Tell me about the worst pain you’ve had this week? (3)
o How much pain were you in last week? (4)
o How much pain were you in the last time we saw each other? (5)
o Did you have pain or headaches last week Bruce? (6)
o I want to know how you are feeling and about your pain. Tell me about the last week Bruce. (7)
o Other: (8) ____
o I do not want to respond (9)

Display This Question:

If Which of the following methods would you use to assess his pain? (select all that apply) = Ask Bruce to rate his pain using a standardized rating tool (e.g., the Faces Pain Scale)

Q50 What standardized rating tool would be your first choice?

____

Q51 Would you consider Bruce’s psychosocial history (e.g., family circumstances, life stressors) when assessing (and potentially when re-assessing) his pain?
o Yes - If yes, how would you use this information (i.e., how would it influence your assessment)? (1) ____
o No - If no, please explain why not? (2) ____

o I do not want to respond (3)

Q52 Would you consider Bruce’s psychosocial history (e.g., family circumstances, life stressors) when deciding on a pain management strategy for him?
o Yes - If yes, how would you use this information (i.e., how would it influence your assessment)? (1) ____
o No - If no, please explain why not? (2)
____
o I do not want to respond (3)

Q53 Would you consider that Bruce’s psychosocial history (e.g., family circumstances, life stressors, maltreatment history) may influence the way in which he communicates his pain?
o Yes (4)
o Maybe (5)
o No (6)

End of Block: Scenario 2

Start of Block: Knowledge and attitudes

Q54 Please indicate if the following statements are true or false.

Q55 Vital signs are always reliable indicators of the intensity of a patient’s pain.
o True (1)
o False (2)
o I do not want to respond (3)

Q56 Because their nervous system is underdeveloped, children under two years of age have decreased pain sensitivity and limited memory of painful experiences.
o True (1)
o False (2)
o I do not want to respond (3)

Q57 Patients who can be distracted from pain usually do not have severe pain.
o True (1)
o False (2)
o I do not want to respond (3)

Q58 Patients may sleep in spite of severe pain.
o True (1)
o False (2)
o I do not want to respond (3)

Q59 Children are more likely to try to suppress their pain than exaggerate it.
o True (1)

o False (2)
o I do not want to respond (3)

Q60 Children with a history of maltreatment are more likely to experience chronic pain than children without a history of maltreatment.
o True (1)
o False (2)
o I do not want to respond (3)

Q61 Pediatric patients (children) should be encouraged to endure as much pain as possible before using an opioid.
o True (1)
o False (2)
o I do not want to respond (3)

Q62 Children with a history of maltreatment are at an increased risk of having their pain intensity underestimated in a medical setting.
o True (1)
o False (2)
o I do not want to respond (3)

Q63 Children less than 11 years old cannot reliably report pain so clinicians should rely solely on the parent's assessment of the child's pain intensity.
o True (1)
o False (2)
o I do not want to respond (3)

Q64 Children with a history of maltreatment are more likely to exaggerate their pain.
o True (1)
o False (2)
o I do not want to respond (3)

Q65 Patients' spiritual beliefs may lead them to think pain and suffering are necessary.
o True (1)
o False (2)
o I do not want to respond (3)

Q66 After an initial dose of opioid analgesic is given, subsequent doses should be adjusted in accordance with the individual patient's response.
o True (1)
o False (2)
o I do not want to respond (3)

Q67 Giving patients sterile water by injection (placebo) is a useful test to determine if the pain is real.

o True (1)
o False (2)
o I do not want to respond (3)

Q68 Children with a history of maltreatment are more likely to exhibit behaviours that express pain.
o True (1)
o False (2)
o I do not want to respond (3)

Q69 If the source of the patient’s pain is unknown, opioids should not be used during the pain evaluation period, as this could mask the ability to correctly diagnose the cause of pain.
o True (1)
o False (2)
o I do not want to respond (3)

Q70 Children with a history of maltreatment are at a greater risk of developing substance misuse problems as a result of chronic pain.
o True (1)
o False (2)
o I do not want to respond (3)

Q71 Children are more likely to suppress their pain than exaggerate it but only if they have a history of maltreatment.
o True (1)
o False (2)
o I do not want to respond (3)

Q72 Children make poor judgements of their own pain and cannot convey their pain levels reliably.
o True (1)
o False (2)
o I do not want to respond (3)

Q73 Children with a history of maltreatment may experience post-traumatic stress symptoms which may subsequently lead to pediatric chronic pain.
o True (1)
o False (2)
o I do not want to respond (3)

Q74 Having a history of maltreatment can influence a child's emotional expression; however, this does not impact the communication of their pain.
o True (1)
o False (2)

o I do not want to respond (3)

Q75 The frequency of victimization (i.e., number of times they have experienced abuse) a child experiences has a cumulative effect on their pain intensity.
o True (1)
o False (2)
o I do not want to respond (3)

Q76 Having a history of maltreatment does not influence pediatric pain expression or assessment.
o True (1)
o False (2)
o I do not want to respond (3)

Q77 If a child's expressions of pain are significantly restricted in comparison to their presenting condition(s), it is valid to suspect a history of maltreatment (and potentially trauma).
o True (1)
o False (2)
o I do not want to respond (3)

End of Block: Knowledge and attitudes

Start of Block: Multiple Choice

Q78 For the following questions please indicate which answer is the most correct.

Q79 The most likely reason a patient with pain would request increased doses of pain medication is:
o The patient is experiencing increased pain. (1)
o The patient is experiencing increased anxiety or depression. (2)
o The patient is requesting more staff attention. (3)
o The patient's requests are related to addiction. (4)
o I do not want to respond (5)

Q80 The most accurate judge of the intensity of a child's pain is:
o The treating physician (1)
o The patient's primary nurse (2)
o The patient (3)
o The pharmacist (4)
o The patient's spouse or family (5)
o I do not want to respond (6)

Q81 Which of the following describes the best approach for cultural considerations in caring for a child in pain:
o There are no longer cultural influences in Canada due to the diversity of the population. (1)
o Cultural influences can be determined by an individual’s ethnicity (e.g., Asians are stoic, Italians are expressive, etc). (2)
o Patients should be individually assessed to determine cultural influences. (3)
o Cultural influences can be determined by an individual’s socioeconomic status (e.g., children from blue collar families report more pain than children from white collar families). (4)
o I do not want to respond (5)

Q82 When assessing an infant for pain, the pain management physician/nurse should recognize that:
o A lack of a physiologic or behavioral response means a lack of pain. (1)
o If something causes pain in an adult, it can cause pain in an infant. (2)
o The parent's observations should not be included in the patient's assessment of pain. (3)
o The Wong-Baker FACES Scale is an appropriate assessment tool. (4)
o I do not want to respond (5)

Q83 If you use an acute pain assessment measure for a child with chronic pain, you would expect to see that:
o Pain scores would be equally as high as a child experiencing acute pain at the same intensity level. (1)
o Pain scores would be lower because chronic pain isn’t as intense as acute pain. (2)
o Pain scores would be lower because chronic pain patients might have more covert expressions of their pain. (3)
o Pain scores would be equally as high because acute measures of pain are not influenced by the patient's pain expression. (4)
o I do not want to respond (5)

End of Block: Multiple Choice

End of Survey
